# Taking on Typhoid: Eliminating Typhoid Fever as a Global Health Problem

**DOI:** 10.1093/ofid/ofad055

**Published:** 2023-06-02

**Authors:** Jessie Chen, Jessica E Long, Kirsten Vannice, Tanya Shewchuk, Supriya Kumar, A Duncan Steele, Anita K M Zaidi

**Affiliations:** Enteric and Diarrheal Diseases, Global Health, Bill & Melinda Gates Foundation, Seattle, Washington, USA; Enteric and Diarrheal Diseases, Global Health, Bill & Melinda Gates Foundation, Seattle, Washington, USA; Enteric and Diarrheal Diseases, Global Health, Bill & Melinda Gates Foundation, Seattle, Washington, USA; Enteric and Diarrheal Diseases, Global Health, Bill & Melinda Gates Foundation, Seattle, Washington, USA; Enteric and Diarrheal Diseases, Global Health, Bill & Melinda Gates Foundation, Seattle, Washington, USA; Enteric and Diarrheal Diseases, Global Health, Bill & Melinda Gates Foundation, Seattle, Washington, USA; Enteric and Diarrheal Diseases, Global Health, Bill & Melinda Gates Foundation, Seattle, Washington, USA

**Keywords:** elimination, typhoid, typhoid conjugate vaccines

## Abstract

Typhoid fever is a significant global health problem that impacts people living in areas without access to clean water and sanitation. However, collaborative international partnerships and new research have improved both knowledge of the burden in countries with endemic disease and the tools for improved surveillance, including environmental surveillance. Two typhoid conjugate vaccines (TCVs) have achieved World Health Organization prequalification, with several more in the development pipeline. Despite hurdles posed by the coronavirus disease 2019 pandemic, multiple TCV efficacy trials have been conducted in high-burden countries, and data indicate that TCVs provide a high degree of protection from typhoid fever, are safe to use in young children, provide lasting protection, and have the potential to combat typhoid antimicrobial resistance. Now is the time to double down on typhoid control and elimination by sustaining progress made through water, sanitation, and hygiene improvements and accelerating TCV introduction in high-burden locations.

Typhoid, caused by *Salmonella enterica* serovar Typhi, is a neglected disease that affects the most vulnerable populations, including those without access to safe water, improved sanitation, and hygiene (WASH) infrastructure. In 2019 alone, typhoid caused an estimated 110 000 deaths and 8.1 million disability-adjusted life-years (DALYs) [1]. However, modeling and meta-analyses suggest that these numbers have steadily declined over the past decade as access to WASH infrastructure and medical care in some geographies has improved, decreasing from an estimated 140 000 deaths and 10.3 million DALYs in 2010. There is further cause for optimism as well; over the past few years, there have been significant developments in both our understanding of disease burden through enhanced surveillance efforts and the development of typhoid conjugate vaccines (TCVs) with robust efficacy and effectiveness data. Additionally, we now have novel tools to further refine our understanding of burden in low- and lower-middle-income countries (LMICs) and to assess progress against typhoid burden [1, 2].

The typhoid vaccine development pipeline has experienced robust progress. There are currently 2 World Health Organization (WHO)–prequalified TCVs, with more products expected to enter the market in the next 1–2 years. TCV clinical protection has been demonstrated in diverse settings, and vaccine introduction in several countries has been achieved due to an energized global public health ecosystem with collaborative manufacturers, enabling partnerships with the Typhoid Vaccine Acceleration Consortium (TyVAC) [3] and the Coalition against Typhoid (CaT) [4], supportive WHO policy and the 2017 recommendation for programmatic TCV use [5], Gavi funding [6], and engaged country partners.

However, it is well recognized that routine immunization programs have experienced unprecedented setbacks due to the severe acute respiratory syndrome coronavirus 2 (SARS-CoV-2) pandemic, resulting in childhood vaccination coverage experiencing the largest decrease in ∼30 years [7, 8]. The WHO has recommended that TCV be deployed through the routine schedule, typically at 9 months, and there have been concerns that its introduction would be hampered due to these circumstances. Nevertheless, although coronavirus disease 2019 (COVID-19) prioritization and COVID-related disruptions initially presented challenges to the vaccine's rollout, the ecosystem of partners has successfully supported smoother and more rapid TCV decision-making and introduction than expected [9]. In less than a year after the first TCV received WHO prequalification, it was deployed for outbreak response in Pakistan and subsequently introduced into Pakistan’s routine immunization program. Several other countries in Africa and Asia have followed suit, and many more are currently gathering evidence and assessing priorities for the decision-making process and Gavi application.

Although the relative prioritization of typhoid immunization fluctuates in countries’ public health agendas, ongoing surveillance studies show that typhoid continues to substantially contribute to morbidity in LMICs. Thanks to many efforts, today we have the tools to address this disease of the most vulnerable and impoverished communities. Now is the time for the global health community to double down on TCV vaccination efforts while maintaining momentum for improved access to WASH. This paper lays out the current estimated typhoid burden, surveillance efforts in place, new data on TCVs, recent and upcoming national introductions, and the reasons for optimism in the global efforts toward typhoid control.

## BURDEN

**Figure 1.**
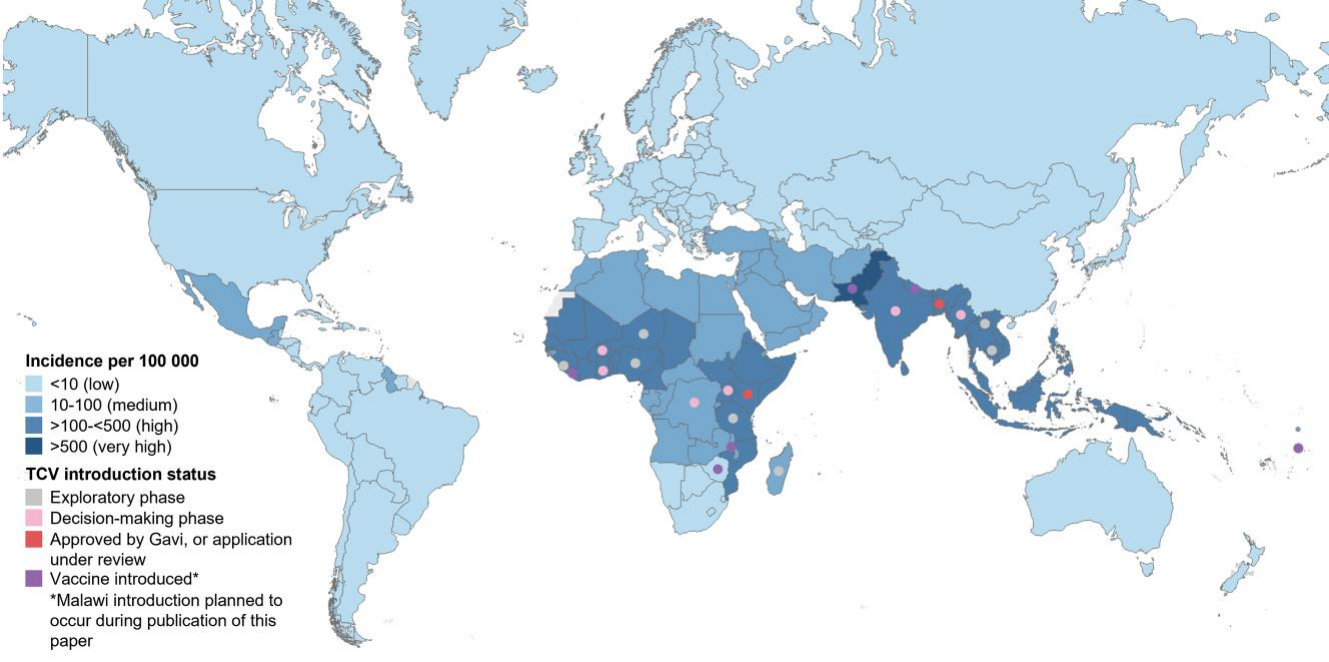
IHME estimated incidence of typhoid in 2019 and TCV introduction status as of April 2023 [1] (PATH representatives, email communication, April 28, 2023). Abbreviations: IHME, Institute for Health Metrics and Evaluation; TCV, typhoid conjugate vaccine.

Typhoid burden has historically been difficult to measure, largely due to the necessity for blood culture for laboratory diagnosis and the paucity of other reliable laboratory tests for appropriate confirmation. Previous estimates of global burden have relied on extrapolation from a limited set of studies [10], but recent investments in regional surveillance have provided more accurate estimates of incidence and mortality, particularly in many African and South Asian countries. As previously outlined [11], there have been several surveillance studies in recent years in areas thought to have endemic typhoid: the Typhoid Fever Surveillance in Africa Program (TSAP) from 2010 to 2014 [12], Severe Typhoid Fever Surveillance in Africa (SETA) established from 2016 [13, 14], the Surveillance for Enteric Fever in Asia Project (SEAP) from 2016 to 2019 [15], Surveillance of Enteric Fever in India (SEFI) [16, 17], and the Wellcome Trust–funded Strategic Typhoid Alliance Across Africa and Asia (STRATAA) [16, 17]. These studies have contributed to a better understanding of the high burden in Asia and revealed that contrary to prior belief, typhoid is also a significant public health problem in many African settings. The latest Global Burden of Disease estimates show the largest typhoid burden in South Asia, Western Sub-Saharan Africa, and Eastern Sub-Saharan Africa, with 76 600, 10 000, and 8100 estimated deaths in 2019, respectively [1]. Estimated typhoid incidence rates show a similar geographic pattern (Figure 1). However, there is considerable year-to-year variation shown between studies, and the inconsistencies in methodologies used to estimate incidence underscore the need for further primary data collection and standardization of incidence rate calculation [2]. Children aged 5–14 years bear a significant portion of typhoid-related morbidity and mortality (with an estimated 46 800 deaths in 2019), so studies including this population are key [1].

High disease burden is one of the most important factors impacting a country's decision to introduce a particular vaccine. The availability of typhoid burden data from surveillance studies and concerns about high rates of antimicrobial resistance (AMR) enabled the WHO Strategic Advisory Group of Experts (SAGE) on Immunizations decision to recommend programmatic TCV use, prioritizing countries with the highest overall burden or AMR threat [5]. Local vaccination strategies are still recommended to be formed with the local typhoid epidemiology and surveillance data quality in mind. Unfortunately, the absence of blood culture capacity in many typhoid-endemic regions means that disease burden remains poorly understood, both regionally and nationally [18]. It is therefore critical that countries and organizations with surveillance capacity continue to monitor typhoid burden and that new tools and approaches be made available to support typhoid burden estimation in those areas that currently have little awareness of burden.

### New Tools to Evaluate Typhoid Burden

In countries or subnational regions where blood culture capacity is lacking, prevalence of *S.* Typhi in the environment, specifically in sewage or environmental water contaminated with human fecal matter such as open drains or rivers, could provide a clue about typhoid infections in the population. Environmental surveillance (ES) methods have been developed for typhoid surveillance and are being validated in the field in Malawi, India, and Ghana [19], as well as in Fiji and Nigeria. ES for typhoid is expected to enable an estimation of whether a location that lacks blood culture has high, medium, or low burden of typhoid. Put together with data on the prevalence of *Salmonella*-associated intestinal perforations in the population and modeled estimates of typhoid burden based on neighboring countries and regions, such data are expected to contribute to an economic rationale to aid decision-making for TCV introduction [20]. Environmental samples enable surveillance of many thousands of people, making it cost-effective compared with individual-level testing [21]. Yet the methods are constrained to areas with confluent wastewater or drainage networks and less applicable in sparsely populated areas. Nonetheless, ES has been used to inform vaccine use against polio [22, 23] and estimate vaccine impact [24] of inactivated poliovirus vaccine. SARS-CoV-2 ES has been used to inform nonpharmaceutical interventions such as geographically targeted increases in access to clinical testing (see case studies in [25]) and to detect emerging variants [26, 27].

Additionally, novel methods in serosurveillance can provide enteric fever seroincidence estimates in countries lacking blood culture–based disease incidence estimates (see [28–30] for further details). The tool is based on an enzyme-linked immunosorbent assay that detects antibodies to *Salmonella*-specific hemolysin E antigen and has been validated with dried blood spots, which are easier to collect and store than serum samples [28]. Planning a population-representative serosurvey is often a challenging undertaking, but countries may plan to leverage stored samples from recently conducted serosurveys that were aimed at gauging prevalence of antibodies to SARS-CoV-2. If access to reagents, equipment, and training for the tool can be provided, seroincidence may provide a way forward for countries to generate additional evidence for TCV introduction decision-making.

WHO guidelines for polio ES to supplement poliovirus surveillance and to inform catch-up vaccine campaigns [31] and use of seroprevalence data to inform hepatitis B vaccine introduction at birth [32] are available and may be models for the development of guidelines for the implementation and interpretation of typhoid ES and serosurveillance.

## NEW DATA ON TYPHOID CONJUGATE VACCINES

### Broadening Efficacy Data

Tremendous progress continues to be made generating policy-relevant data on TCV efficacy, immunogenicity, safety, durability, and co-administration with other vaccines. Typbar-TCV, a Vi-TT conjugate vaccine and the first TCV receiving WHO prequalification, was approved in 2018 using data from a controlled human infection model (CHIM) showing efficacy of 87.1% (95% CI, 47.2%–96.9%) against clinically relevant typhoid fever [33]. Since then, the TyVAC consortium and other partners have generated substantial data on the efficacy and effectiveness of Typbar-TCV through clinical trials and leveraging routine introductions. Before the pandemic, data from a 12-month interim analysis of a randomized clinical trial (RCT) in Nepal estimated vaccine efficacy at 81.6% (95% CI, 58.8%–91.8%; *P* < .001) against *S.* Typhi bacteremia in children 9 months to 16 years [34]. These were the first available data on the efficacy of the Vi-TT vaccine and showed comparability to the findings of the original CHIM study. Despite the enormous challenges presented by the COVID-19 pandemic, data from the full 2-year Nepal trial, as well as data from Pakistan, Bangladesh, India, and Malawi, have emerged (Table 1). The suite of studies in high-burden countries shows that TCVs have the potential to consistently achieve a high level of protection.

**Table 1.** New Evidence on Clinical Protection of Typbar-TCV

Location	Study Type	Duration of Follow-up	Age Range Vaccinated	Vaccine Efficacy (95% CI), %
Nepal [35]	Ph3 RCT	24 mo	9 mo to 16 y	79 (62–89)
Malawi [36]	Ph3 RCT	24 mo	9 mo to 12 y	81 (64–90)
Bangladesh [37]	Cluster RCT	17 mo	9 mo to 16 y	85 (66–91)
Navi Mumbai, India [38]	Case–control	N/A	9 mo to 14 y	80 (53–92)
Hyderabad, Pakistan [39]	Cohort	24 mo	6 mo to 10 y	95 (93–96)
Karachi, Pakistan [40]	Case–control	N/A	6 mo to 15 y	72 (34–88)

Abbreviations: RCT, randomized controlled trial; TCV, typhoid conjugate vaccine.

There have been several critical questions for TCVs, including the following: How well do they protect the youngest children? Are the vaccines safe? Can they be co-administered with other vaccines? What is the duration of protection? And is there any indirect protection? A study on Vi-TT from Bangladesh showed high-level protection for children ≤2 years, with no significant difference from older age groups [37]. Vi-TT can also be safely co-administered with childhood Expanded Programme on Immunization (EPI) vaccines usually administered at 9 months of age, without vaccine interference, lending support to TCV inclusion among routine immunization schedules [41, 42]. Given the potential of newer vaccines to be added to the childhood EPI schedule in various countries (eg human papilloma virus vaccine HPV] and malaria vaccines such as RTS, S), there may be future studies with expanded safety data. As for duration of protection, the data thus far support TCV use as a single-dose vaccine for at least 3 years [43], although there are immunological data suggesting that seroconversion may persist in at least a subset of vaccinated children up to 7 years [44]. Further confirmation will be required to assess the necessity of a booster dose. Finally, the evidence for indirect protection by Vi-TT is very modest, but analysis has not been conducted on a study sufficiently powered to detect this [37].

### Second Prequalified Typhoid Conjugate Vaccine

TyphiBEV, a Vi-CRM_197_ vaccine developed by Biological E, Ltd, India, received WHO prequalification in December 2020 based on safety and immunogenicity established in a phase 2/3 study [45]. TyphiBEV demonstrated noninferiority compared with Typbar-TCV after 42 days, and there were no differences in safety profiles between the 2 vaccines [46]. Currently a phase 4 clinical trial is ongoing to examine the impact of TyphiBEV introduction in South India (VEVACT, NCT05500482). Additional studies of vaccine effectiveness are anticipated as the vaccine is introduced in routine programs.

There is a robust development pipeline that includes 2 TCVs, SK Bioscience's and PT Bio Farma's Vi-DT (diphtheria toxoid) vaccines. Both have recently achieved national licensure and are pursuing WHO prequalification, and phase 3 data from SK Bioscience's Vi-DT shows noninferiority to Vi-TT for safety as well as immunogenicity. There are also other TCV candidates at earlier stages of development [47]. Having multiple TCV vaccines reaching WHO prequalification allows for increased security in vaccine supply and availability and contributes to a “healthy market” as defined by Gavi, The Vaccine Alliance.

## TCV INTRODUCTIONS

Despite the COVID-19 pandemic, there has been significant progress in introducing TCVs (Figure 1). In Asia, Pakistan led the way by being the first country to incorporate TCV into the national immunization schedule in 2019 after initial targeted use in an outbreak setting against an extensively drug-resistant (XDR) strain of *S.* Typhi [39], and the scaled introduction was recently completed, reaching millions of children nationwide [48]. In 2021, Samoa held a mass vaccination campaign and became the first non-Gavi country to introduce TCV [49]. That same year, Liberia became the first African country to introduce TCV into its routine immunization schedule using regional data of disease burden and limited clinical data [50]. It was followed shortly by Zimbabwe in 2021, also responding to a multidrug-resistant (MDR) outbreak of typhoid fever [51], and Nepal in April 2022 based on high disease burden [52]. Malawi has received approval from Gavi and is set to introduce TCV in the first half of 2023 [45].

There is accelerating momentum for national introductions in many other countries with multiple new National Immunization Technical Advisory Group (NITAG) recommendations in countries with a recognized burden of typhoid. The Indian NTAGI recommended TCV introduction in June 2022 [53], while the Bangladesh and Kenya NITAGs made similar recommendations and have applied to Gavi for vaccine subsidy support [54]. Several other countries across Asia and Africa are currently considering the introduction of TCV to their national immunization schedules. Vaccine effectiveness data from countries that have already introduced TCV, coupled with regional and modeling data and possibly novel surveillance tools, should provide more evidence for surrounding countries to consider implementation. Ongoing studies such as the Typhoid Conjugate Vaccine Effectiveness in Ghana (TyVEGA) study [55] may provide information on TCV indirect effects in a locale with lower transmission intensity, in comparison with the modest effects observed in a high-density population in Bangladesh. As noted above, various data points have triggered decisions to introduce TCVs, including hospital data on typhoid perforations, disease modeling, emerging antimicrobial resistance [56], and cost-effectiveness information. While each country has a unique set of considerations, these resources, in addition to economic cost of illness studies [57, 58], provide a comprehensive set of data upon which countries considering new TCV introduction may draw.

The WHO has previously recognized that vaccines play a role in the battle against AMR, and in 2022 the WHO published an action framework that calls out the value of TCV to prevent infections and reduce antimicrobial use, specifically noting the estimated impact in Pakistan [59]. Recent work has also noted the potential of TCV to avert 342 000 deaths (95% prediction interval, 135 000–1.5 million) in Gavi-eligible countries [60]. Given the plethora of novel typhoid-related developments, the SAGE committee met in April 2022 to assess recent and upcoming data on typhoid burden and vaccines [61]. *S.* Typhi was recommended to be included as a pathogen of interest in future sessions on AMR, with the role of genomic studies to be assessed. SAGE reaffirmed existing recommendations on TCV use and noted data supportive of expansion of the age range of use up to 65 years, although official WHO guidelines for appropriate use up to age 45 remain in place, with catch-up campaigns advised up to 15 years due to disease burden and program feasibility in that age range [5]. At present, Gavi provides funding for routine immunization and catch-up campaigns up to 15 years of age. Population mobility is also recommended as a consideration for vaccination strategy formulation [61].

## MOVING FORWARD

### Areas of Further Research

The next phase of enhancing global control efforts is to drive sustained momentum for TCV use in endemic settings; with this in mind, a number of strategic areas of research are underway to provide the requisite data. First, as previously stated, more easily accessible data on disease burden at the local or regional level may help inform TCV rollout strategy and prioritization. New environmental surveillance methods being studied (outlined above in “New Tools to Evaluate Typhoid Burden”) may provide a more detailed picture of local variations in disease burden. In cases where blood culture surveillance data are not available, pediatric surgical ward sentinel surveillance for cases of intestinal perforation in children, indicative of *Salmonella* infection [62], may be used as a proxy indicator of typhoid burden and assist countries in TCV introduction decision-making.

Second, more data are needed to confirm the impact of TCV on antimicrobial-resistant and XDR Typhi strains. Drug resistance is becoming an increasingly critical concern [63], and modeling studies suggest that introduction of TCV during routine immunization at age 9 months along with catch-up campaigns up to age 15 years can markedly reduce the impact of AMR typhoid [60]. Further empirical data will be useful in understanding the full potential of TCVs, particularly for prioritizing introductions to areas with high AMR. The ongoing Pakistan TCG Impact Study (ITRIPP) is aimed at understanding the impact of TCV on long-term transmission of AMR in Pakistan.

Finally, research into the feasibility of typhoid elimination is an ongoing area of study. Humans are the only known reservoir for *S.* Typhi, and the availability of effective tools in the form of vaccines, diagnostics, and environmental solutions such as sewage treatment and chlorination of drinking water together provide a possible means of eliminating typhoid. Current research is focused on ongoing efforts to improve surveillance and diagnostic tools and focus on typhoid control in the immediate future.

### New Tools and Their Applications

Beyond TCVs, additional instruments are in development to support the goal of typhoid control. It is imperative that we use new environmental surveillance and serosurveillance tools effectively to ensure that countries have the evidence needed to make TCV introduction decisions in a timely manner. Gavi is assessing available surveillance tools and will be evaluating potential low-cost tools that can then be made available to countries to enable them to determine their typhoid burden. Gavi is also funding research to develop new diagnostics that will help guide clinical treatment. As progress is made toward typhoid control, chronic *S.* Typhi carriers will become an important focus of new development. Chronic infection occurs in 3%–5% of individuals following acute clinical or subclinical infection, and these carriers are likely causing new cases decades after infection [64]. Research in Chile is being conducted to improve identification of chronic carriers [65], and new methods of targeting treatment to these carriers will be critical to achieve typhoid elimination.

### Sustaining Momentum and Driving Impact

**Figure 2.**
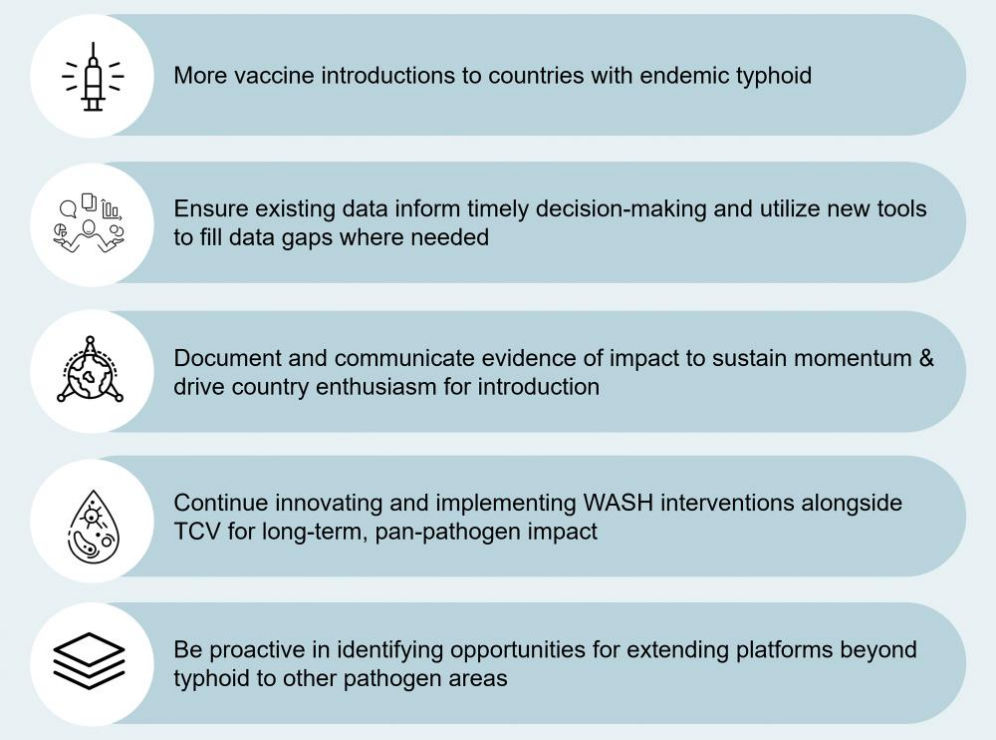
A roadmap for eliminating typhoid as a global health problem. Abbreviations: TCV, typhoid conjugate vaccine; WASH, safe water, improved sanitation, and hygiene.

Given the success of TCV development, the primary focus moving forward is on sustained momentum for TCV use in endemic settings. Many partners in the typhoid control community, including Gavi, are aligned on the goals to accelerate TCV introduction and to ensure sufficient supply of low-cost, prequalified TCV from multiple manufacturers for delivery to Gavi-eligible countries and LMICs. Driving these milestones is the recognition that sustained impact will rely on generation of more data, documentation and communication of evidence, and a holistic view that incorporates other pathogen areas (Figure 2). While vaccines are an extremely important tool, we recognize that there is a broad range of interventions that are important to the ultimate success of typhoid control, including improved diagnostic tools and WASH infrastructure. There may also be novel, combination vaccines that can be developed, including a bivalent enteric fever vaccine to target both *S.* Typhi and *S*. Paratyphi A. Recognizing the changing landscape of climate change, increasing migration and urbanization, and the increasing MDR of *Salmonella* strains, there is a global public health imperative to accelerate our efforts toward the control of enteric fever.

## CONCLUSIONS

Important progress has been made in the fight against typhoid thanks to a strong partner ecosystem and country engagement that has seen reductions in burden in key regions, as well as advancements on many fronts. The WHO prequalification of 2 TCVs, with several more in the pipeline, provides an optimistic picture for vaccine supply security in the future. TCVs have been introduced in 5 countries, and there is a growing number of countries that are anticipated to incorporate TCV into their routine immunization programs in the coming years. Building out surveillance to achieve wider geographical coverage of burden data and improved representation of rural areas will be crucial to inform future TCV introduction strategies, and new diagnostic tests will allow for improvements in both burden data and proper clinical management. Continued strong partnerships, both globally and at the country level, will underpin our ability to maintain momentum on what has been a neglected disease. The combined strategy of leveraging TCV while continuing to develop new tools will move us closer to achieving the goal of typhoid elimination as a global health problem.
